# (1*RS*,2*RS*,3*RS*)-1,2-Dimeth­oxy-3-methyl-2-phenyl-1-(2-thien­yl)cyclo­propane

**DOI:** 10.1107/S1600536809009441

**Published:** 2009-03-19

**Authors:** Laura Torre-Fernández, Marcos G. Suero, Santiago García-Granda

**Affiliations:** a Departamento de Química Física y Analítica, Facultad de Química, Universidad de Oviedo, C/ Julián Clavería, 8, 33006 Oviedo, Spain; b Departamento de Química Orgánica e Inorgánica, Facultad de Química, Universidad de Oviedo, C/ Julián Clavería, 8, 33006 Oviedo, Spain

## Abstract

In the title compound, C_16_H_18_O_2_S, a new *cis*-1,2-dimethoxy­cyclo­propane, the two meth­oxy groups are in a *cis* configuration and in *trans* positions with respect to the H atom and the phenyl and thienyl rings on the cyclo­propyl group. The mol­ecular packing is dominated by weak inter­molecular C—H⋯O inter­actions, allowing the formation of zigzag chains propagating parallel to the *c* axis. The dihedral angle between the aromatic rings is 86.12 (8)°.

## Related literature

For related literature on the chemistry, see: Lebel *et al.* (2003 ▶). For a general overview of the biological implications of cyclo­propane-related derivatives, see: de Meijere *et al.* (2003 ▶). For their occurrence, see: Wessjohann *et al.* (2003 ▶).
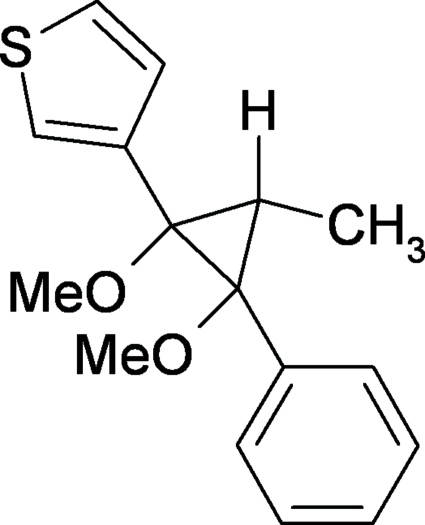


## Experimental

### 

#### Crystal data

C_16_H_18_O_2_S
*M* *_r_* = 274.36
Monoclinic, 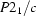

*a* = 12.9924 (3) Å
*b* = 9.7194 (2) Å
*c* = 14.7960 (3) Å
β = 128.395 (1)°
*V* = 1464.37 (6) Å^3^
*Z* = 4
Cu *K*α radiation
μ = 1.92 mm^−1^
*T* = 293 K
0.56 × 0.35 × 0.28 mm

#### Data collection

Oxford Diffraction Nova diffractometer
Absorption correction: refined from Δ*F* (*XABS2*; Parkin *et al.*, 1995 ▶) *T*_min_ = 0.330, *T*_max_ = 0.581
7070 measured reflections
2830 independent reflections
2435 reflections with *I* > 2σ(*I*)
*R*_int_ = 0.021

#### Refinement

*R*[*F* ^2^ > 2σ(*F* ^2^)] = 0.055
*wR*(*F* ^2^) = 0.192
*S* = 1.16
2830 reflections
172 parameters
H-atom parameters constrained
Δρ_max_ = 0.47 e Å^−3^
Δρ_min_ = −0.52 e Å^−3^

### 

Data collection: *CrysAlis CCD* (Oxford Diffraction, 2006 ▶); cell refinement: *CrysAlis RED* (Oxford Diffraction, 2006 ▶); data reduction: *CrysAlis RED*; program(s) used to solve structure: *SIR92* (Altomare *et al.*, 1994 ▶); program(s) used to refine structure: *SHELXL97* (Sheldrick, 2008 ▶); molecular graphics: *ORTEP-3 for Windows* (Farrugia, 1997 ▶); software used to prepare material for publication: *WinGX* (Farrugia, 1999 ▶).

## Supplementary Material

Crystal structure: contains datablocks global, I. DOI: 10.1107/S1600536809009441/cs2110sup1.cif

Structure factors: contains datablocks I. DOI: 10.1107/S1600536809009441/cs2110Isup2.hkl

Additional supplementary materials:  crystallographic information; 3D view; checkCIF report

## Figures and Tables

**Table 1:** Hydrogen-bond geometry (Å, °)

*D*—H⋯*A*	*D*—H	H⋯*A*	*D*⋯*A*	*D*—H⋯*A*
C16—H16⋯O2^i^	0.93	2.55	3.469 (3)	172

Symmetry code: (i) 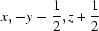.

**Fig. 1.**
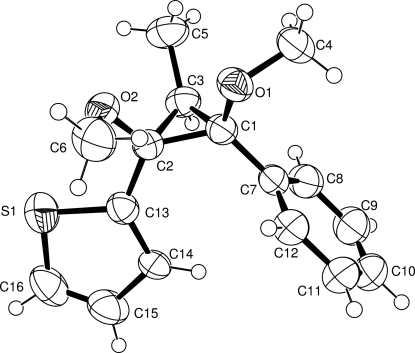
A view of the title compound with displacement ellipsoids drawn at 50% probability level.

## supplementary crystallographic information

### Comment

The cyclopropane ring is a quite common subunit of natural products isolated from plants, fungi, and microorganisms (Wessjohann, *et al.* 2003). Many of these natural products show biological activity, and some of them have found applications as drugs or insecticides (de Meijere *et al.*, 2003). Classical chemical synthesis of cyclopropane derivatives include the halomethyl-metal mediated cyclopropanation of olefins, the transition-metal-catalyzed carbene-transfer reaction from diazo compounds, and the nucleophilic-addition/ring-closing sequence (Lebel *et al.* 2003). A new method for the synthesis of *cis*-1,2-dimethoxycyclopropane through the cyclopropanation of lithium ketone enolates with Fischer carbene complex will be published elsewhere. The molecular structure of the title compound is shown in Fig. 1. There are no unusual bonding features. O atoms of the two methoxy groups are in *cis* position to each other and in *trans* positions with the C3 hydrogen atom, and point away from the phenyl and from thienyl rings on the cyclopropyl group, respectively. The molecular packing is dominated by the weak intermolecular interaction C16—H16 ··· O2 allowing the formation of zig-zag chains roughly parallel to the *c* crystallographic axis and perpendicular to the *b* axis.

### Experimental

Lithium enolate of 2-acetylthiophene was prepared by treatment of a solution of the corresponding ketone (1.2 mmol, 151 mg) and lithium diisopropylamide (1.2 mmol, 0.39 *M*, 3.1 ml) at 195 K for 30 mins. Pentacarbonyl(1-methoxy-1-phenylmethylene)-chromium (1 mmol, 312 mg) in THF (10 ml) was added over lithium enolate solution at 195 K. Cooling bath was removed and the reaction mixture allowed to warm up to 273 K and stirred for a further 45 mins, concentrated in high vacuum, redisolved in Et_2_O (10 ml) and cooled to 195 K. TfOMe (2.0 mmol, 224 µ*L*) was added dropwise to the mixture. After 5 mins, cooling bath was removed and the reaction mixture was stirred for 30 min while allowing the temperature to reach 273 K. The reaction mixture was quenched with NH_4_Cl (20 ml). The resulting mixture was diluted with hexanes/ethyl acetate, 10/1 (110 ml) and subjected to air oxidation under sunlight. After 2–12 h the suspension was filtered through Celite and extracted with diethyl ether (3 *x* 10 ml). The organic layers were combined, dried over anhydrous Na_2_SO_4_ and concentrated *in vacuo*. The crude product was purified by flash column chromatography on silica gel (hexanes/ethyl acetate, 20/1) to yield the title compound (277 mg, 77%) as a 1:1 diastereoisomer mixture of the *all-S* (1,2,3) and *all-R* forms.

### Refinement

At the end of the refinement the highest peak in the electron density was 0.47 e Å ^-3^. The deepest hole was -0.52 e Å ^-3^.

### Crystal data

**Table:**

C_16_H_18_O_2_S	*F*(000) = 584
*M**_r_* = 274.36	*D*_x_ = 1.244 Mg m^−^^3^
Monoclinic, *P*2_1_/*c*	Cu *K*α radiation, λ = 1.54184 Å
Hall symbol: -P 2ybc	Cell parameters from 5163 reflections
*a* = 12.9924 (3) Å	θ = 4.3–74.9°
*b* = 9.7194 (2) Å	µ = 1.92 mm^−^^1^
*c* = 14.7960 (3) Å	*T* = 293 K
β = 128.395 (1)°	Prism, colourless
*V* = 1464.37 (6) Å^3^	0.56 × 0.35 × 0.28 mm
*Z* = 4	

### Data collection

**Table:**

Oxford Diffraction Nova diffractometer	2830 independent reflections
Radiation source: Nova (Cu) X-ray Source	2435 reflections with *I* > 2σ(*I*)
graphite	*R*_int_ = 0.021
Detector resolution: 8.2640 pixels mm^-1^	θ_max_ = 75.0°, θ_min_ = 4.3°
ω scans	*h* = −15→15
Absorption correction: part of the refinement model (Δ*F*) (*XABS2*; Parkin *et al.*, 1995)	*k* = −12→11
*T*_min_ = 0.330, *T*_max_ = 0.581	*l* = −16→18
7070 measured reflections	

### Refinement

**Table:**

Refinement on *F*^2^	Primary atom site location: structure-invariant direct methods
Least-squares matrix: full	Secondary atom site location: difference Fourier map
*R*[*F*^2^ > 2σ(*F*^2^)] = 0.055	Hydrogen site location: inferred from neighbouring sites
*wR*(*F*^2^) = 0.192	H-atom parameters constrained
*S* = 1.16	*w* = 1/[σ^2^(*F*_o_^2^) + (0.1086*P*)^2^ + 0.4259*P*] where *P* = (*F*_o_^2^ + 2*F*_c_^2^)/3
2830 reflections	(Δ/σ)_max_ < 0.001
172 parameters	Δρ_max_ = 0.47 e Å^−^^3^
0 restraints	Δρ_min_ = −0.52 e Å^−^^3^

### Special details

**Table:**

Geometry. All e.s.d.'s (except the e.s.d. in the dihedral angle between two l.s. planes) are estimated using the full covariance matrix. The cell e.s.d.'s are taken into account individually in the estimation of e.s.d.'s in distances, angles and torsion angles; correlations between e.s.d.'s in cell parameters are only used when they are defined by crystal symmetry. An approximate (isotropic) treatment of cell e.s.d.'s is used for estimating e.s.d.'s involving l.s. planes.

### Fractional atomic coordinates and isotropic or equivalent isotropic displacement parameters (Å^2^)

**Table:**

	*x*	*y*	*z*	*U*_iso_*/*U*_eq_	
S1	0.20782 (8)	−0.21540 (8)	0.43464 (7)	0.0761 (3)	
O1	0.21967 (15)	−0.00779 (16)	0.12428 (13)	0.0513 (4)	
O2	0.17975 (16)	−0.20993 (15)	0.22182 (15)	0.0549 (4)	
C2	0.24847 (18)	−0.0958 (2)	0.29248 (17)	0.0431 (5)	
C14	0.2341 (2)	0.0488 (3)	0.43703 (18)	0.0521 (5)	
H14	0.2479	0.1376	0.4230	0.063*	
C13	0.22814 (19)	−0.0728 (2)	0.37976 (18)	0.0473 (5)	
C7	0.2809 (2)	0.1667 (2)	0.26751 (17)	0.0462 (5)	
C8	0.3982 (3)	0.2360 (3)	0.3460 (2)	0.0597 (6)	
H8	0.4776	0.1894	0.3840	0.072*	
C4	0.2883 (3)	0.0554 (3)	0.0888 (2)	0.0654 (7)	
H4B	0.2447	0.0343	0.0091	0.098*	
H4A	0.3767	0.0211	0.1350	0.098*	
H4C	0.2898	0.1533	0.0982	0.098*	
C1	0.27813 (19)	0.0184 (2)	0.24060 (17)	0.0435 (5)	
C12	0.1637 (2)	0.2390 (3)	0.2108 (2)	0.0566 (6)	
H12	0.0844	0.1941	0.1575	0.068*	
C3	0.38579 (19)	−0.0804 (2)	0.32878 (19)	0.0499 (5)	
H3	0.4492	−0.0403	0.4056	0.060*	
C16	0.2013 (3)	−0.1224 (4)	0.5271 (2)	0.0744 (8)	
H16	0.1892	−0.1606	0.5775	0.089*	
C5	0.4434 (3)	−0.1874 (3)	0.2983 (3)	0.0680 (7)	
H5C	0.5304	−0.1603	0.3283	0.102*	
H5B	0.3894	−0.1960	0.2160	0.102*	
H5A	0.4471	−0.2742	0.3312	0.102*	
C6	0.0420 (3)	−0.1869 (3)	0.1399 (2)	0.0707 (7)	
H6C	0.0000	−0.2683	0.0944	0.106*	
H6A	0.0258	−0.1117	0.0904	0.106*	
H6B	0.0073	−0.1650	0.1795	0.106*	
C9	0.3975 (3)	0.3743 (3)	0.3681 (3)	0.0770 (8)	
H9	0.4764	0.4199	0.4213	0.092*	
C15	0.2154 (3)	0.0112 (3)	0.5202 (2)	0.0725 (8)	
H15	0.2134	0.0758	0.5654	0.087*	
C10	0.2806 (4)	0.4449 (3)	0.3115 (3)	0.0805 (9)	
H10	0.2808	0.5378	0.3265	0.097*	
C11	0.1640 (3)	0.3783 (3)	0.2333 (3)	0.0722 (7)	
H11	0.0851	0.4260	0.1953	0.087*	

### Atomic displacement parameters (Å^2^)

**Table:**

	*U*^11^	*U*^22^	*U*^33^	*U*^12^	*U*^13^	*U*^23^
S1	0.0953 (6)	0.0630 (5)	0.0924 (6)	0.0042 (3)	0.0694 (5)	0.0161 (3)
O1	0.0570 (8)	0.0570 (9)	0.0485 (8)	−0.0101 (7)	0.0369 (7)	−0.0091 (6)
O2	0.0592 (9)	0.0437 (9)	0.0685 (10)	−0.0084 (6)	0.0430 (8)	−0.0110 (7)
C2	0.0444 (10)	0.0407 (10)	0.0474 (10)	−0.0004 (7)	0.0300 (8)	−0.0020 (8)
C14	0.0751 (14)	0.0535 (12)	0.0511 (11)	0.0033 (10)	0.0508 (11)	0.0016 (9)
C13	0.0455 (10)	0.0502 (12)	0.0485 (11)	0.0042 (8)	0.0304 (9)	0.0057 (8)
C7	0.0570 (11)	0.0442 (11)	0.0512 (10)	−0.0057 (9)	0.0404 (10)	−0.0038 (8)
C8	0.0634 (13)	0.0580 (14)	0.0687 (14)	−0.0138 (11)	0.0464 (12)	−0.0144 (11)
C4	0.0861 (17)	0.0669 (16)	0.0714 (15)	−0.0114 (13)	0.0628 (14)	−0.0076 (12)
C1	0.0441 (10)	0.0458 (11)	0.0454 (10)	−0.0042 (8)	0.0302 (8)	−0.0044 (8)
C12	0.0645 (13)	0.0530 (13)	0.0613 (13)	0.0040 (10)	0.0435 (11)	0.0036 (10)
C3	0.0434 (10)	0.0512 (12)	0.0545 (11)	0.0004 (8)	0.0301 (9)	−0.0065 (9)
C16	0.0739 (16)	0.095 (2)	0.0701 (15)	0.0119 (15)	0.0523 (14)	0.0275 (15)
C5	0.0604 (13)	0.0686 (16)	0.0820 (17)	0.0100 (11)	0.0477 (13)	−0.0076 (13)
C6	0.0588 (14)	0.0811 (18)	0.0701 (15)	−0.0210 (12)	0.0389 (13)	−0.0191 (13)
C9	0.104 (2)	0.0571 (15)	0.0944 (19)	−0.0321 (15)	0.0740 (18)	−0.0298 (14)
C15	0.0888 (18)	0.088 (2)	0.0629 (15)	0.0051 (15)	0.0579 (15)	0.0005 (13)
C10	0.134 (3)	0.0452 (13)	0.106 (2)	−0.0082 (16)	0.096 (2)	−0.0102 (14)
C11	0.099 (2)	0.0547 (15)	0.0890 (18)	0.0175 (14)	0.0713 (17)	0.0124 (13)

### Geometric parameters (Å, °)

**Table:**

S1—C16	1.686 (3)	C1—C3	1.521 (3)
S1—C13	1.708 (2)	C12—C11	1.394 (4)
O1—C1	1.407 (2)	C12—H12	0.9300
O1—C4	1.425 (3)	C3—C5	1.504 (3)
O2—C2	1.400 (2)	C3—H3	0.9800
O2—C6	1.424 (3)	C16—C15	1.324 (5)
C2—C13	1.487 (3)	C16—H16	0.9300
C2—C3	1.518 (3)	C5—H5C	0.9600
C2—C1	1.529 (3)	C5—H5B	0.9600
C14—C13	1.428 (3)	C5—H5A	0.9600
C14—C15	1.441 (3)	C6—H6C	0.9600
C14—H14	0.9300	C6—H6A	0.9600
C7—C8	1.389 (3)	C6—H6B	0.9600
C7—C12	1.389 (3)	C9—C10	1.378 (5)
C7—C1	1.490 (3)	C9—H9	0.9300
C8—C9	1.384 (4)	C15—H15	0.9300
C8—H8	0.9300	C10—C11	1.369 (5)
C4—H4B	0.9600	C10—H10	0.9300
C4—H4A	0.9600	C11—H11	0.9300
C4—H4C	0.9600		
			
C16—S1—C13	92.92 (13)	C11—C12—H12	119.7
C1—O1—C4	112.78 (17)	C5—C3—C2	121.32 (19)
C2—O2—C6	113.11 (18)	C5—C3—C1	122.9 (2)
O2—C2—C13	113.14 (17)	C2—C3—C1	60.40 (13)
O2—C2—C3	113.99 (17)	C5—C3—H3	114.0
C13—C2—C3	118.79 (17)	C2—C3—H3	114.0
O2—C2—C1	116.28 (16)	C1—C3—H3	114.0
C13—C2—C1	124.24 (18)	C15—C16—S1	112.6 (2)
C3—C2—C1	59.91 (13)	C15—C16—H16	123.7
C13—C14—C15	108.7 (2)	S1—C16—H16	123.7
C13—C14—H14	125.6	C3—C5—H5C	109.5
C15—C14—H14	125.6	C3—C5—H5B	109.5
C14—C13—C2	131.81 (19)	H5C—C5—H5B	109.5
C14—C13—S1	110.92 (15)	C3—C5—H5A	109.5
C2—C13—S1	116.98 (16)	H5C—C5—H5A	109.5
C8—C7—C12	118.8 (2)	H5B—C5—H5A	109.5
C8—C7—C1	121.7 (2)	O2—C6—H6C	109.5
C12—C7—C1	119.55 (19)	O2—C6—H6A	109.5
C9—C8—C7	120.3 (3)	H6C—C6—H6A	109.5
C9—C8—H8	119.9	O2—C6—H6B	109.5
C7—C8—H8	119.9	H6C—C6—H6B	109.5
O1—C4—H4B	109.5	H6A—C6—H6B	109.5
O1—C4—H4A	109.5	C10—C9—C8	120.4 (3)
H4B—C4—H4A	109.5	C10—C9—H9	119.8
O1—C4—H4C	109.5	C8—C9—H9	119.8
H4B—C4—H4C	109.5	C16—C15—C14	114.8 (3)
H4A—C4—H4C	109.5	C16—C15—H15	122.6
O1—C1—C7	114.01 (17)	C14—C15—H15	122.6
O1—C1—C3	116.44 (17)	C11—C10—C9	120.1 (3)
C7—C1—C3	121.67 (17)	C11—C10—H10	119.9
O1—C1—C2	111.72 (16)	C9—C10—H10	119.9
C7—C1—C2	122.67 (16)	C10—C11—C12	119.9 (3)
C3—C1—C2	59.69 (13)	C10—C11—H11	120.1
C7—C12—C11	120.6 (2)	C12—C11—H11	120.1
C7—C12—H12	119.7		
			
C6—O2—C2—C13	73.1 (2)	C3—C2—C1—O1	108.80 (19)
C6—O2—C2—C3	−147.1 (2)	O2—C2—C1—C7	145.90 (19)
C6—O2—C2—C1	−80.2 (2)	C13—C2—C1—C7	−4.1 (3)
C15—C14—C13—C2	−175.9 (2)	C3—C2—C1—C7	−110.3 (2)
C15—C14—C13—S1	−2.4 (3)	O2—C2—C1—C3	−103.76 (19)
O2—C2—C13—C14	−153.1 (2)	C13—C2—C1—C3	106.2 (2)
C3—C2—C13—C14	69.2 (3)	C8—C7—C12—C11	−0.6 (3)
C1—C2—C13—C14	−2.2 (3)	C1—C7—C12—C11	−179.0 (2)
O2—C2—C13—S1	33.7 (2)	O2—C2—C3—C5	−5.1 (3)
C3—C2—C13—S1	−103.9 (2)	C13—C2—C3—C5	132.3 (2)
C1—C2—C13—S1	−175.39 (15)	C1—C2—C3—C5	−112.6 (2)
C16—S1—C13—C14	1.88 (18)	O2—C2—C3—C1	107.59 (19)
C16—S1—C13—C2	176.44 (17)	C13—C2—C3—C1	−115.1 (2)
C12—C7—C8—C9	0.8 (3)	O1—C1—C3—C5	9.3 (3)
C1—C7—C8—C9	179.2 (2)	C7—C1—C3—C5	−137.9 (2)
C4—O1—C1—C7	62.3 (2)	C2—C1—C3—C5	110.2 (2)
C4—O1—C1—C3	−87.4 (2)	O1—C1—C3—C2	−100.84 (19)
C4—O1—C1—C2	−153.32 (19)	C7—C1—C3—C2	112.0 (2)
C8—C7—C1—O1	−118.9 (2)	C13—S1—C16—C15	−0.8 (2)
C12—C7—C1—O1	59.4 (2)	C7—C8—C9—C10	−0.7 (4)
C8—C7—C1—C3	29.0 (3)	S1—C16—C15—C14	−0.5 (4)
C12—C7—C1—C3	−152.6 (2)	C13—C14—C15—C16	1.8 (4)
C8—C7—C1—C2	101.0 (2)	C8—C9—C10—C11	0.3 (4)
C12—C7—C1—C2	−80.6 (3)	C9—C10—C11—C12	−0.1 (4)
O2—C2—C1—O1	5.0 (2)	C7—C12—C11—C10	0.3 (4)
C13—C2—C1—O1	−145.00 (18)		

### Hydrogen-bond geometry (Å, °)

**Table:**

*D*—H···*A*	*D*—H	H···*A*	*D*···*A*	*D*—H···*A*
C16—H16···O2^i^	0.93	2.55	3.469 (3)	172

Symmetry codes: (i) *x*, −*y*−1/2, *z*+1/2.
